# Environmental influences on and antimicrobial activity of the skin microbiota of *Proceratophrys boiei* (Amphibia, Anura) across forest fragments

**DOI:** 10.1002/ece3.5949

**Published:** 2020-01-07

**Authors:** Ananda B. Assis, Catherine R. Bevier, Cristine Chaves Barreto, Carlos Arturo Navas

**Affiliations:** ^1^ Department of Physiology Institute of Bioscience University of São Paulo São Paulo Brazil; ^2^ Department of Biology Colby College Waterville ME USA; ^3^ Graduate Program in Genomic Sciences and Biotechnology Catholic University of Brasília Brasília Brazil

**Keywords:** amphibian, antimicrobials, environmental microbes, fragmented forests, metabolites, skin microbiota

## Abstract

The composition of the skin microbiota of amphibians is related to the biology of host species and environmental microbial communities. In this system, the environment serves as a microbial source and can modulate the hosted community. When habitats are fragmented and the environment disturbed, changes in the structure of this microbial community are expected. One important potential consequence of fragmentation is a compromised protective function of the microbiota against pathogenic microorganisms. In this study, the skin microbiota of the amphibian *Proceratophrys boiei* was characterized, evaluated for relationships with environmental variables and environmental sources of microbial communities, and its diversity evaluated for frog populations from fragmented and continuous forests. In addition, the antimicrobial activity of this skin community was studied in frogs from both forest types. Culture methods and 16S rRNA high‐throughput gene sequencing were used to characterize the microbial community and demonstrated that the skin microbiota of *P. boiei* is more closely related to the soil microbial communities than those inhabiting water bodies or fragment matrix, the unforested area around the forested fragment. The microbial diversity and abundance of *P*. *boiei* skin microbiota are different between continuous forests and fragments. This community is correlated with environmental variables, especially with temperature of microhabitat and distance to human dwelling. All individuals of *P. boiei* harbored bacteria capable of inhibiting the growth of pathogenic bacteria and different strains of the pathogenic fungus *Batrachochytrium dendrobatidis*, and a total of 27 bacterial genera were detected. The results of this study indicate that the persistence of populations of this species will need balanced and sustained interactions among host, microorganisms, and environment.

## Graphical abstract

Macro and microenvironmental effects on the skin microbiota of the frog species *Proceratophrys boiei*. The skin microbiota of the frog *P. boiei* presents variation between preserved forests and fragments. All individuals of *P. boiei* harbor bacteria capable of inhibiting the growth of pathogenic bacteria and different strains of the fungus *Batrachochytrium dendrobatidi*.

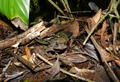

## 1 INTRODUCTION

The colonization of the skin of amphibians by symbiotic microorganisms seems to occur through contact among and between individuals and environmental microbial communities (Assis, Barreto, & Navas, 2017; Baas‐Becking, 1934). Because the environment is both source and regulator of the skin communities, habitat alteration can affect microorganism‐host interactions (Becker, Fonseca, Haddad, & Prado, 2009). Responses to habitat alteration in the amphibian skin microbiota are expected because microorganisms display great ability to adjust to shifts in external conditions at both the cellular and population levels (Becker, Longo, Haddad, & Zamudio, 2017). Accordingly, the fragmentation of landscape, which changes the environmental conditions, provides an opportunity to evaluate consequent changes in the microbial diversity of amphibians. Fragmented habitats vary greatly in both biotic and abiotic features because the process of fragmentation is a result of altered physical and local human activities (Becker & Harris, 2010). In addition, because fragmentation also affects amphibian behavior and ecology (Becker et al., 2015; Bennett & Saunders 2010), it may induce simultaneous changes in amphibian biology and the dynamics of microorganisms on amphibian skin.

The cutaneous microbiota may complement the immune system as a protective barrier against pathogens such as *Batrachochytrium dendrobatidis* (*Bd*; Berger, 2001). The mechanisms for such protection are under current investigation, but both the reduction of surface area, where the pathogens can settle, and the production of antimicrobial molecules seem relevant factors (Bjork, O'Hara, Ribes, Coma, & Montoya, 2018). Indeed, such dermal microbial communities could prevent epidemics in wild populations of amphibians (Brockett, Prescott, & Grayston, 2012; Burkey, 2013). The hypothesis that microbial communities on the skin of amphibians may be altered when habitat is fragmented deserves careful testing given the clear relevance of such communities for these vertebrates. Thus, if habitat fragmentation alters the skin microbiota, then fragmentation can indirectly affect individual health and population resilience.

A relevant question is, what factors determine the microbial community profile that can establish over the amphibian skin? In other words, what fraction of an environmental microbiota has the potential to colonize the skin, what elements become a filter, and how do intrinsic and extrinsic factors matter in this context? One line of research suggests relevance of phylogenetic affinity. For example, host species identity explains a substantial portion of variation in the microbial community on the skin across several species (Caporaso et al., 2010). However, the environment is also an important factor in determining and maintaining the structure of these symbiont communities (Baas‐Becking, 1934). Both factors likely matter to differing degrees and may vary with host species, local environmental conditions, and health status of the population.

Dermal microbial communities play a protective role for the amphibian host, but physiological disorders can result when the natural structure of a symbiont community is disturbed (Carey, Cohen, & Rollins‐Smith, 1999). Abundance, for example, is important in the relationship between the resident community and the host and influences the protective role of beneficial microbiota when competing with invasive pathogens (Christin et al., 2004). Furthermore, the biosynthesis of certain bioactive molecules is determined in part by the population density of the productive microorganisms (Clarke & Warwick, 2001). In the case of amphibians, experimental changes in the abundances of cutaneous microbiota lead to greater susceptibility to chytridiomycosis, the disease caused by *Bd* infection (Crawshaw & Fowler, 1993). Therefore, environmental conditions that help maintain the structure of the cutaneous microbiota of amphibians are likely to favor resilience of the host populations. For example, when habitat connectivity is maintained, there is greater similarity in the skin microbiota profile among adjacent amphibian populations (Davidson et al., 2007). Thus, identifying how an amphibian's environment can affect the community structure of the amphibian skin microbiome is fundamental to understanding the consequences of habitat alteration on microorganism–host interaction.

This study addresses the influence of habitat fragmentation on the community of cutaneous microbiota of anurans, and we focus on two assumptions that are supported independently in the literature but have not been addressed concurrently: (a) The microbial community present on the skin of amphibians is composed, at least partially, of bacteria found in surrounding environments (Baas‐Becking, 1934); (b) environmental microbial communities are affected by the changes in environmental variables (DeAngelis et al., 2015; Dunny & Winans, 1999) related to fragmentation processes (Eaton, Clesceri, Greenberg, & Franson, 1995). We designed and conducted a single‐species study with *Proceratophrys boiei*, a frog distinctive to the Atlantic Forest of Brazil and whose cutaneous microbiota has been characterized (Funk, Greene, Corn, & Allendorf, 2005). Our aims were to (a) identify the microbial environmental sources for *P. boiei* skin microbiota; (b) compare the diversity, composition, and abundance of *P. boiei* skin microbiota between frog populations in fragmented and continuous forests; (c) evaluate the influence of environmental variables on the composition and abundance of the skin microbiota; and 4) investigate the antimicrobial capacity of the cutaneous microbiota of *P. boiei*.

## 2 MATERIALS AND METHODS

### 2.1 Overall design

The relationship between host and environmental microbial communities was tested by comparing the composition and abundance of skin microbial communities, categorized as “skin,” with those from habitats, namely “bodies of water” and “soil,” where frogs were found. A fourth habitat was categorized as soil outside fragments and called “matrix,” a term consistent with landscape ecology. Samples from frogs and habitats from two forest types, “continuous” and “fragment” were compared, and, by definition, did not contain “matrix” habitat. Microbial diversity was assessed by 16s rRNA high‐throughput sequencing. Finally, the antimicrobial capacity of skin microbiota isolated from individual *P. boiei* was tested against the growth of pathogenic bacteria and *Bd* strains, by culturing the skin microbiota and using metabolites in challenge assays.

### 2.2 Study area

**Figure 1:**
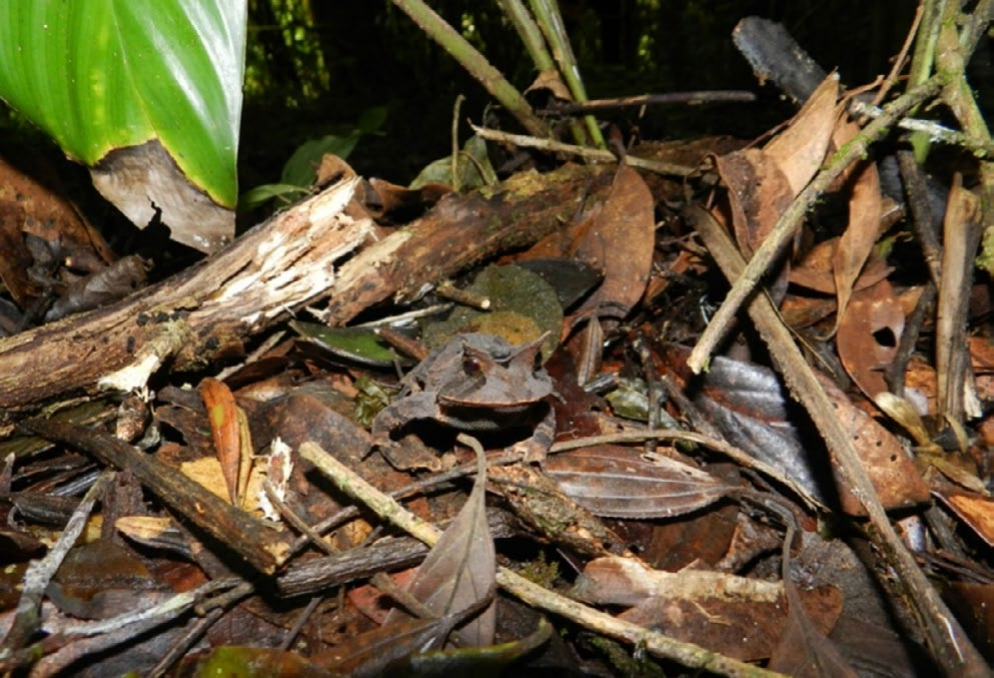
*Proceratophrys boiei* in its microhabitat

**Figure 2:**
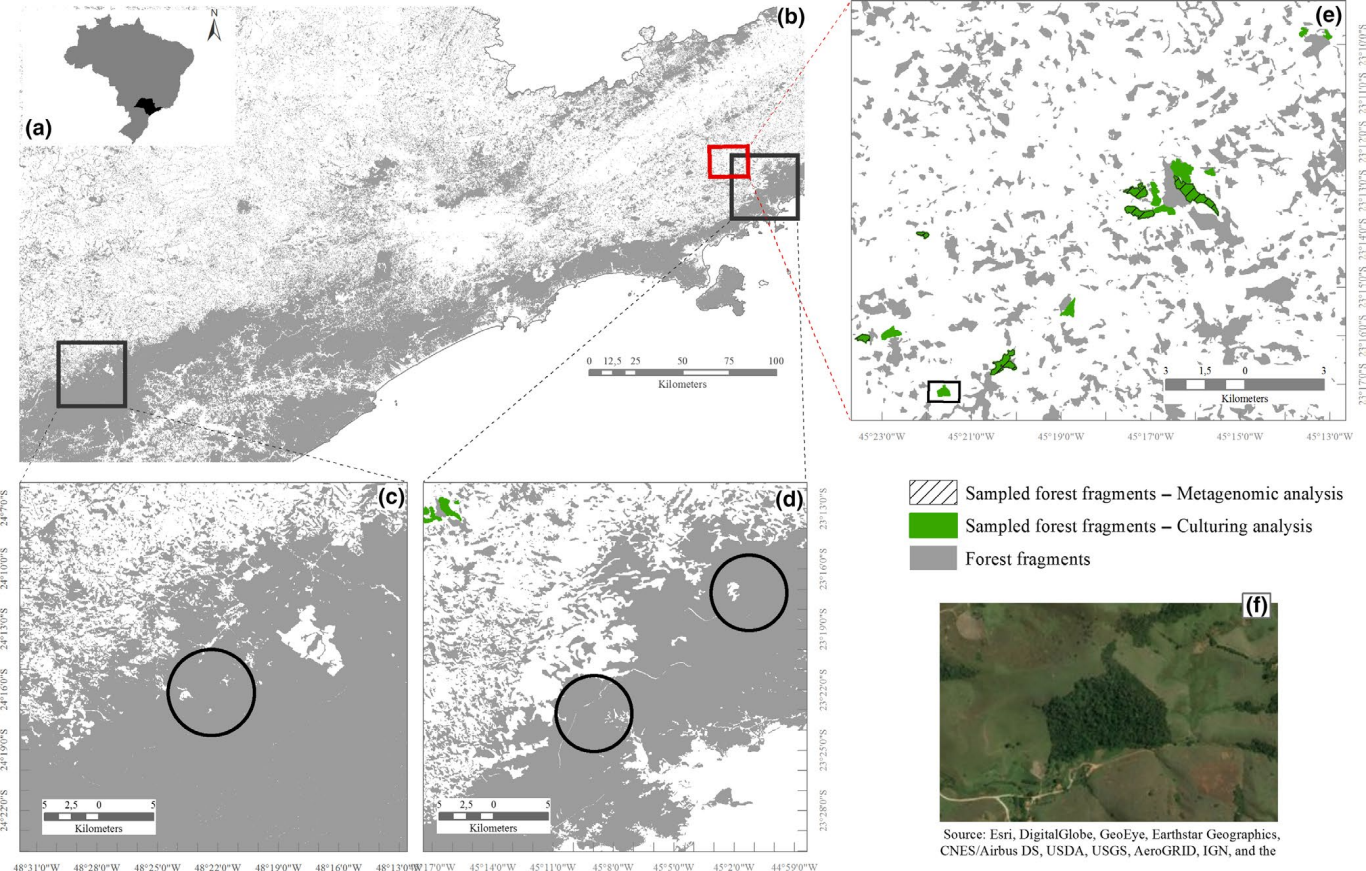
Sampling sites. (a) Location of study area in São Paulo state (Brazil); (b) Study area of continuous and fragmented forests; (c) Site of Paraíso farm and Intervales State Park continuous forests; (d) Sites of Serra do Mar State Park, nucleus of Santa Virginia (left.) and Cunha (right) continuous forests; (e) Distribution of 15 sampled fragment forests of São Luís do Paraitinga and Redenção da Serra municipalities, in the State of São Paulo. All green fragments were sampled for culturing analysis, and green hatched fragments were sampled for metagenomic analysis. (f) Image of one fragment with pasture matrix (square amplified from e)

Fifteen forest fragments were selected, ranging from 60.7 ha to 3.1 ha, based on documented reports for presence of *Proceratophrys boiei* (Cycloramphidae, Figure 1). These remnants are in the municipalities of São Luís do Paraitinga and Redenção da Serra in the State of São Paulo. The landscape is intensely fragmented and composed of a mosaic of scattered forests, pastures, agricultural monocultures of corn and *Eucalyptus* spp., and urban areas (Figure 2b,e,f). The forest of the Serra do Mar State Park, Cunha and Santa Virgínia Nucleus, and the forests of the State Park of Intervales and Fazenda Paraíso EcoLodge were included in this project as reference habitats containing large tracts of intact forest (Figure 2b–d).

### 2.3 Sampling the cutaneous microbiota

Skin microbiota of *P. boiei* were sampled during the species' reproductive season, specifically December 2011 to March 2012, and October 2012 to February 2013. Up to ten individuals at each site were sampled. Frogs were found by actively searching along trails, streams, or short trails made for sampling. Sterile gloves were used to capture frogs, which were placed individually in a Whirl‐Pak® (Nasco) sterile bag and transported to a local laboratory. Up to 2 hr of travel time between capture and swabbing was allowed across all sampling sites. Once at the laboratory, each frog was washed in two sterile Milli‐Q water baths using about 100 ml (Gerritsen, Smidt, Rijkers, & Vos, 2011). A sterile swab was passed five times each along the snout‐vent length of the dorsum and the venter, placed in 1 ml GTE buffer (20% Glucose – 1 M Tris‐HCL pH 7.4 – 0.5 M EDTA pH 8.0), and stored immediately in a freezer at − 20°C. Animals were returned to the site of capture within 24 hr. The procedures performed in this study were approved by the Ethics Committee on Animal Use of the Institute of Biosciences, University of São Paulo under protocol 129/2011.

### 2.4 Sampling of environmental microbial communities

Samples of soil microbial communities were collected at each site where an individual *P. boiei* was captured; five swab samples were taken from soil and vegetation surfaces within a 50‐cm‐diameter circle centered on the capture site of the frog. Microbial communities of water bodies were sampled primarily from streams adjacent to where frogs were captured. One liter of water was collected from each stream and filtered through a cellulose acetate filter with 0.20 μm pore (Sartorius Stedim Biotech) using a vacuum pump. Surfaces from the fragment matrix (defined here as the unforested area around the forested fragment), considered habitat unsuitable for a given community or species studied (Hall, 1999), were sampled using the same procedure for sampling the habitat near each frog. The fragment matrix was sampled 50 m from the fragment edge and into cleared areas, taking as reference point the specific location where an individual frog was collected. All swab samples were placed in sterile tubes containing GTE buffer (20% Glucose – 1 M Tris‐HCL pH 7.4 – 0.5 M EDTA pH 8.0) and stored in a freezer at − 20°C.

### 2.5 DNA extraction and 16s rRNA high‐throughput sequencing

DNA of the microbial communities of all microenvironments: soil, water, matrix, and skin was extracted using the Power Soil™ DNA Isolation kit (MoBio Laboratories). The DNA of all individuals for each site and microhabitat (soil, matrix, skin, and water) were pooled. The V3 and V4 regions (Hammer, Harper, & Ryan, 2001) of the 16s rRNA gene were amplified using the primers Bakt_341F (CCTACGGGNGGCWGCAG) and Bakt_805R (GACTACHVGGGTATCTAATCC). Amplification and preparation of the libraries were completed at Macrogen Korea (Geumcheon‐gu), and sequencing was done using Illumina MiSeq (2 × 300 bp), 100 k reads/sample, at the same local.

### 2.6 Measurement of environmental variables

When a frog was captured, air and soil temperatures, relative humidity, and soil pH were measured in the immediate vicinity. Water pH, water temperature, dissolved oxygen, conductivity, total dissolved solids, and salinity were measured and recorded for any nearby water body. Dataloggers (HOBO models UA00164 and U10003) for temperature and humidity measurements were placed in microhabitats where *P. boiei* were found to characterize the microclimate. These loggers were programmed to take eight readings per day. Temperature and relative humidity recordings in the sampling areas were made between December 2011 and October 2013.

### 2.7 Antimicrobial activity

#### 2.7.1 Culturing the cutaneous microbiota

The same sampling technique described above was used for collecting microbiota to culture, and each swab was placed in 1 ml of sterile saline (NaCl 0.9%) solution. Each sample was vortexed at high speed for 1 min, diluted 100‐fold in 0.9% NaCl, and cultured on R2A agar (Difco). Cultures were incubated at room temperature (22°–30°C) for 48–72 hr. The total number of colonies for each frog was determined per morphotype, visualized with the aid of a magnifying glass in a colony counting apparatus (Quimis), and the standard method of “heterotrophic plate count” (HCP) was followed (Harris, James, Lauer, Simon, & Patel, 2006).

#### 2.7.2 Identification of isolated bacteria using Sanger sequencing

Each unique morphotype was isolated and identified by sequencing the 16S rRNA gene using three primers, the standard primers 27 F, 1452 R (Hernandez‐Agreda, Gates, & Ainsworth, 2017) and the internal primer 338 F, to obtain the nearly full sequence. Each reaction was composed of 1 U of Top Taq Master Mix (Qiagen), 1.5 mM of MgCl_2_, 0.2 mM of each dNTP, and 0.2 μM of each primer. The amplification program was as follows: 3 min of denaturation at 95°C, 30 cycles of 30 s at 94°C, 30 s at 52°C, 1:40 min at 72°C for extension, and one final cycle at 72°C for 7 min (Hoff, Frye, & Jacobson, 1984). Amplification was assessed by agarose gel electrophoresis, and the product obtained was purified using the GeneJET PCR Purification kit (Thermo Fisher Scientific #K0702), following the manufacturer's recommendations. Nucleotide sequences were analyzed at Universidade Católica de Brasília. The sequences obtained from the isolated bacteria were edited in the Bioedit program (Knights et al., 2011) to remove the low‐quality bases and obtain a contig of approximately 1,300 bp. The classification was completed in the Ribosomal Database Program (RDP) using the Classifier and Sequence match tools with 90% of confidence threshold (Konopka, 2009).

#### 2.7.3 Bacterial pathogens assay

The antimicrobial activity of *P. boiei* skin bacteria against counterparts known to cause amphibian diseases (Kueneman et al., 2014; Lam, Walke, Vredenburg, & Harris, 2010; Lam, Walton, & Harris, 2011; Lammel, Nüsslein, Tsai, & Cerri, 2015; Lane, 1991; Lauer et al., 2007) was investigated using the cross‐streak method (Longo & Zamudio, 2017). Briefly, an isolated morphotype was inoculated onto one side of a petri dish (90 × 15 mm) containing R2A agar (Difco) and incubated at 22°C until the entire surface was seeded visibly by colonies. Perpendicular traces of reference bacterial strains were then made on the opposite side of the plate. Cultures were incubated for 24 hr, and antimicrobial activity was scored as the absence of growth of the test bacteria in the region close to the growth mass of each isolated bacteria. The optimal growth temperature of each pathogen was used: *Micrococcus luteus* (ATCC 7468) and *Enterobacter aerogenes* (ATCC 113048) were incubated at 30°C, while *Klebsiella pneumoniae* subsp. (ATCC 4352), *Staphylococcus aureus* (ATCC 14458), *Staphylococcus epidermidis* (ATCC 12228*), Enterococcus faecalis* (ATCC 10100), *Proteus vulgaris* (ATCC 13315), *Enterobacter aerogenes* (ATCC 113048), *Escherichia coli* (ATCC 10536), *Salmonella enterica* (CT) (ATCC 13076), *Salmonella enterica* (ATCC 6539), *Aeromonas hydrophila* (IOC/FDA 110–36), and *Pseudomonas aeruginosa* (ATCC 15442) were incubated at 37°C.

#### 2.7.4 Chytrid fungus assay

The protocol used to culture, maintain, and harvest *Bd* is widely implemented (Longo & Zamudio, 2017). Two actively growing strains of *Bd*, JEL423 isolated from a treefrog in Panama and CLFT023, isolated from a frog in Brazil, were grown for 1 week on 1% tryptone agar. Zoospores were collected under sterile conditions by flooding the petri dish with 3 ml of 1% tryptone broth and vacuum filtering the suspension over a 20 μm sterile nylon membrane (Spectra/Mesh; Spectrum Laboratory Products). The concentration of zoospores collected for each strain was estimated using a hemocytometer and zoospores stained with 0.4% trypan blue. Dilutions were made to standardize the zoospore concentration to 1 × 10^6^ cells/ml. Metabolites of bacteria isolated from the cutaneous microbiota of *P. boiei* were extracted by inoculating 50 ml of R2A broth at room temperature and growing the culture to the stationary growth phase. Bacterial growth was monitored using optical density (OD) measured with a spectrophotometer at a wavelength of 595 nm. The culture was then centrifuged for 10 min at 5,974 x *g*, the supernatant filtered over a 0.20 μm membrane (Millex, Millipore), and lyophilized. Growth inhibition of *Bd* was tested in vitro following published methods (Longo & Zamudio, 2017). 96‐well plates containing *Bd* zoospores (50 μl) and bacterial metabolites (50 μl) reconstituted with 1 ml of sterile HPLC‐grade water were incubated at 23°C, and OD_492_ was measured on day 0 and day 7. Growth inhibition reported here was calculated based on measurements taken on day 7 using the formula (1 − (experimental inoculum growth (OD)/positive control growth (OD))) × 100.

### 2.8 Statistical analysis

#### 2.8.1 Analysis for 16S rRNA high‐throughput gene sequencing

The Quantitative Insights into Microbial Ecology (Qiime 1.9.1) pipeline was used to process data from high‐throughput 16S rRNA sequencing and obtain the quality control of the paired‐end sequences, the operational taxonomic units (OTUs) and their taxonomic attributions, according to the open reference protocol (Loudon et al., 2014). An identity of 97% was based on the Greengenes database from May 2013. Matrices with unweighted and weighted UniFrac distance metrics, generated in Qiime, were used as input for the Anosim (Similarity Analysis) test to compare microbial communities. Principal Coordinate Analysis (PCoA) ordinations were then generated within the Phyloseq package in R. The SourceTracker tool in Qiime (Madigan, Martinko, Dunlap, & Clark, 2009) was used to estimate the Bayesian probability for every OTU in the *P. boiei* skin microbiota to be derived from soil, water, or matrix communities. Analysis of alpha diversity was performed after rarefaction at 59,091 sequences per sample (minimum sampling depth) and estimated by Chao 1 and Shannon diversity indices to evaluate the diversity of OTUs in the skin, water, soil, and matrix samples. Unpaired *t* tests, with Welch's correction for unequal variances, were performed to compare the diversity between continuous and fragmented forests.

The relationships between environmental variables and microbial communities were evaluated through the Testing matched resemblance matrices (RELATE). In this analysis, UniFrac matrices for biotic data and Euclidean distance matrix for abiotic data were used. The Biota‐Environmental STepwise matching procedure (BEST) was carried out to identify which best combination of environmental variables can explain the patterns of the microbial communities (McKenzie, Bowers, Fierer, Knight, & Lauber, 2012). All these analyses were run in the statistical program Primer v7. Correlations were calculated with the Spearman rank correlation coefficient (*ρ*). Alpha was set at .05 in all analyses.

#### 2.8.2 Analysis for culture dependent

The alpha diversity of antimicrobial skin bacteria was compared between forest types using the diversity *t* test in Past 3.26 software, which is based on the Shannon and Simpson formula (Metzger, 2001). The percentage of *Bd* growth inhibition was compared between *Bd* strains and forest type using a Mann–Whitney *U* test.

## 3 RESULTS

### 3.1 Microbial environmental sources

**Figure 3:**
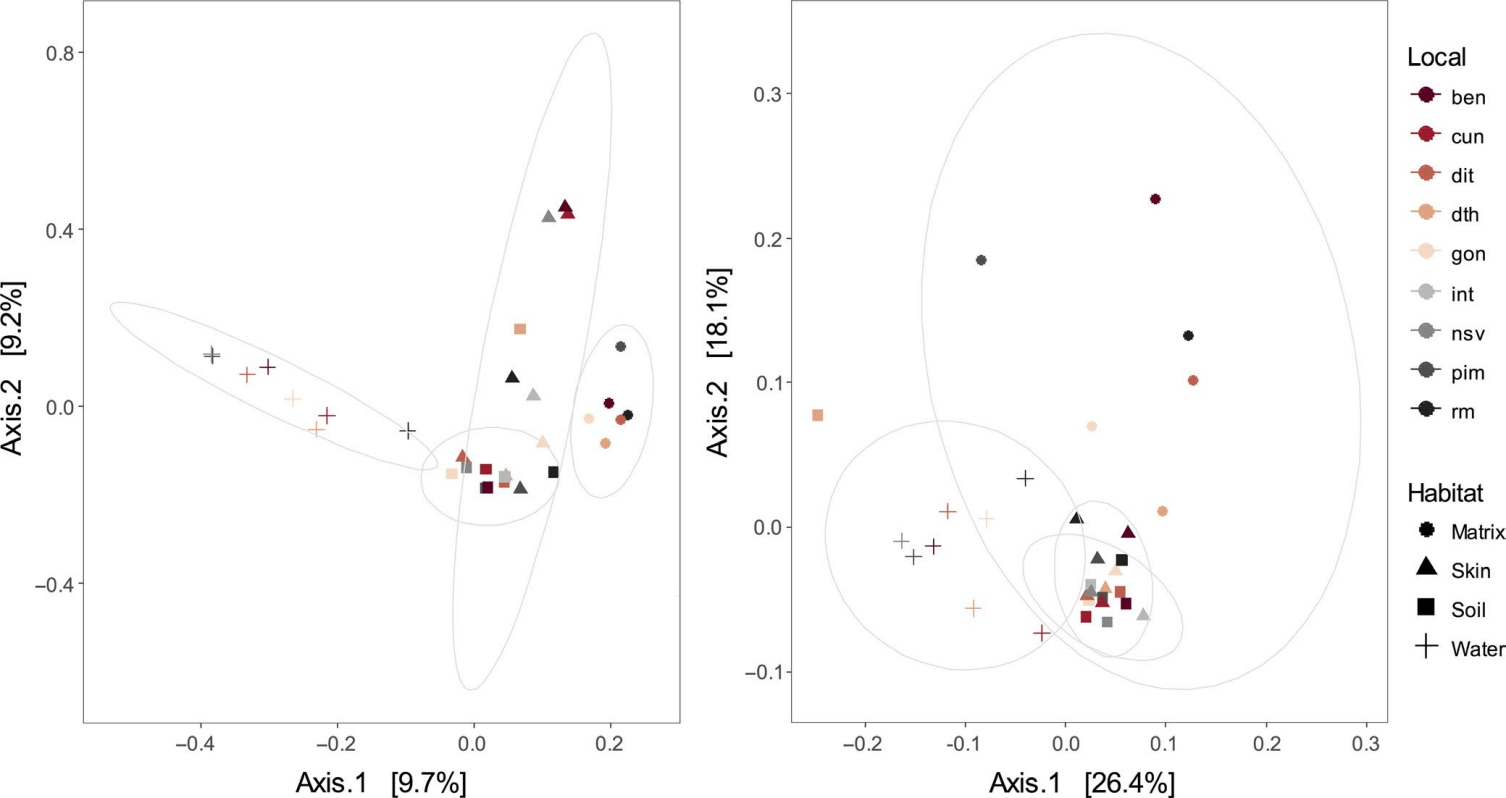
Principal coordinate analysis (PCoA) plots with unweighted (a) and weighted (b) UniFrac distance matrix among bacterial communities across habitats: fragment matrix, skin microbiota, forest soil, and water body are indicated by different symbols. Ellipses were drawn with confidence level at 0.95. Image was generated using the Phyloseq package

**Figure 4:**
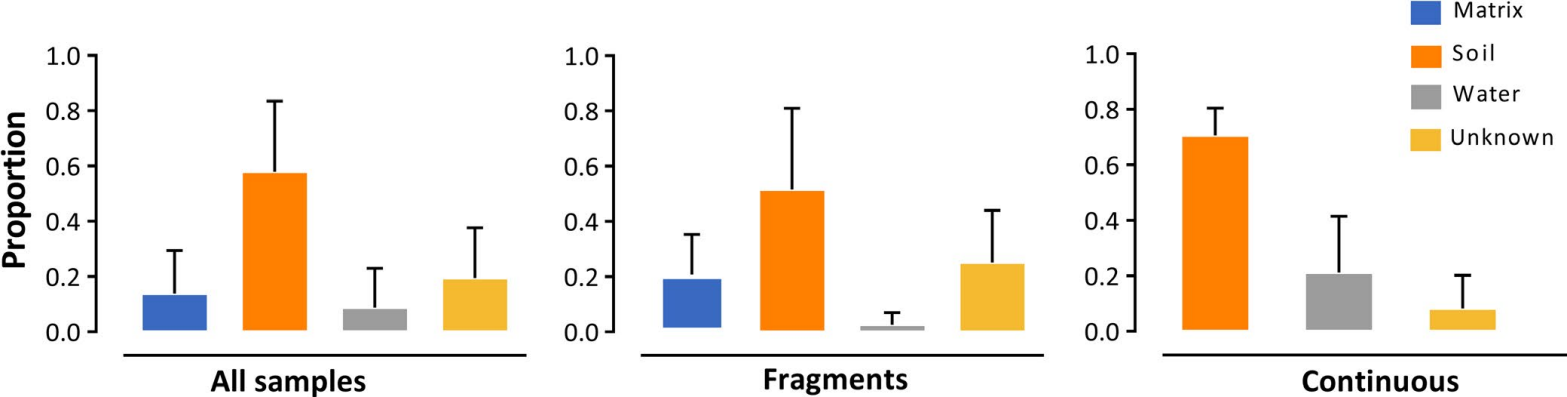
SourceTracker‐based estimates of the proportion of bacterial OTU in *Proceratophrys boiei* skin microbiota derived from matrix, soil, and water samples. “All samples” includes both fragments and continuous forest samples. “Unknown” samples indicate uncertain classification by the SourceTracker algorithm

The four microhabitats, including: soil, water, skin, and matrix, differed in microbial community composition (ANOSIM, *R* = 0.471, *p* = .001/unweighted UniFrac distance), even when the relative abundances were considered (ANOSIM, *R* = 0.398, *p* = .001/ weighted UniFrac distance). These differences were confirmed by pairwise tests that revealed a high degree of significance and robust *R* values. However, the soil–skin pair had a small *R* value (close to zero) indicating that the composition of the communities was strongly overlapping even though the differences were significant (Table 1). PCoA ordinations reflect that *P. boiei* skin microbiota had a stronger relationship with soil than water or matrix bacterial communities (Figure 3a,b). Furthermore, the SourceTracker analysis showed that the primary environmental source for the *P. boiei* skin microbiota were soil communities, which corresponds to a mean of 57.9 ± 25% for all samples, 51.6 ± 29% for fragment samples, and 70.6 ± 9% for continuous forest samples (Figure 4).

**Table 1:** Analysis of similarities (ANOSIM) pairwise test by habitats samples, based on unweighted UniFrac metric

Groups	*R* value	*p* value
Water – Soil	.698	.001
Water – Skin	.481	.001
Water – Matrix	.678	.001
Soil – Skin	.151	.042
Soil – Matrix	.614	.002
Skin – Matrix	.426	.003

### 3.2 Habitat quality and microbial communities

**Figure 5:**
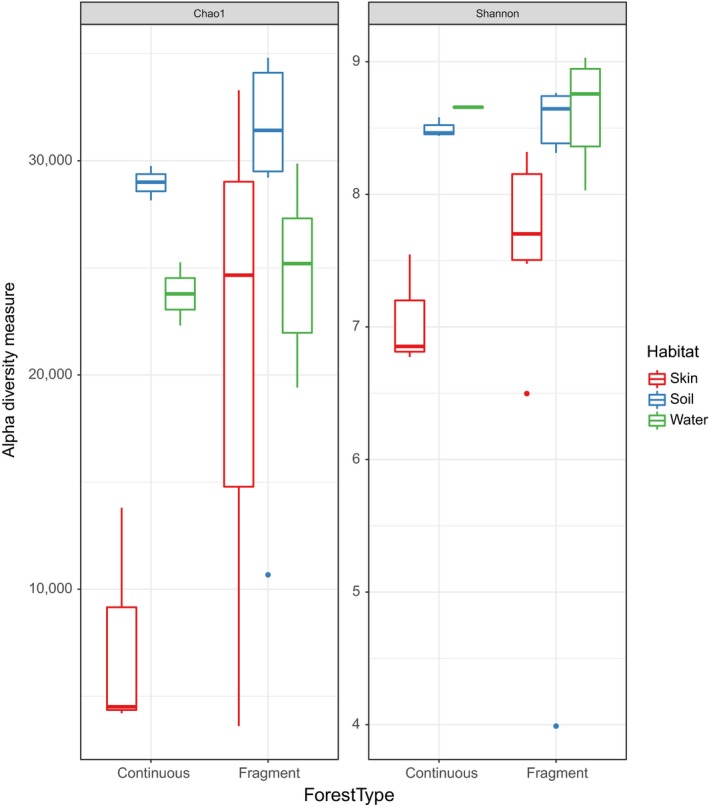
Alpha diversity between fragmented and continuous forests from the habitats of *Proceratophrys boiei* skin, soil, and water. Metrics and plots were generated using the Phyloseq package based on OTU rarefied data. Within habitats, only boxes with same letters are significantly different. No inter‐habitat comparisons were performed in this analysis

The highest diversity index values were found in the microbial communities of the forest fragments (Figure 5). There were no differences in the environmental microbial diversity (Shannon) or richness (Chao 1) between the landscapes (unpaired *t* test with Welch's correction, *p*> .05), but differences were significant for the microbial communities of *P. boiei* skin evaluated by the Chao 1 index (unpaired *t* test with Welch's correction, *p* = .047). The same pattern was observed for unrarefied data; only skin microbiota samples were different between forest types (Chao 1 index; unpaired *t* test with Welch's correction, *p* = .045).

**Figure 6:**
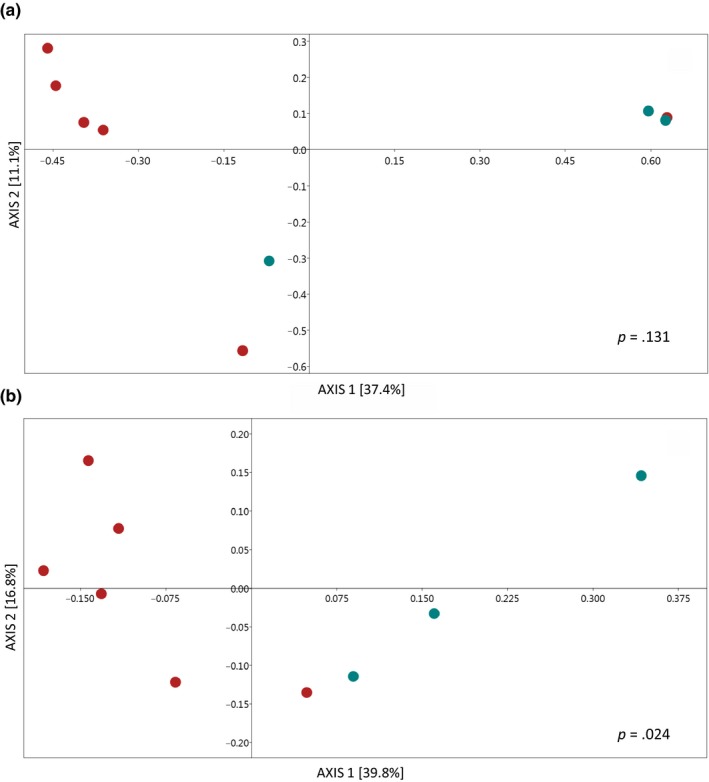
Principal coordinate analysis (PCoA) plots with unweighted (a) and weighted UniFrac (b) distance matrices of *Proceratophrys boiei* skin microbiota

There were no differences in the composition of bacterial communities (unweighted UniFrac) between fragmented and continuous forests for water (ANOSIM, *R* = 0.031, *p* = .393), soil (*R* = −0.111, *p* = .655), and skin (*R* = 0.216, *p* = .131). However, when the relative abundance was considered (weighted UniFrac), there was significant difference in the skin microbiota of *P. boiei* between these two forest types (*R* = 0.438, *p* = .024, Figure 6).

### 3.3 Microbial communities and abiotic variables

The relationship between microbial communities, based on weighted and unweighted UniFrac distance metrics, and environmental variables, using a Euclidean distance matrix, was evaluated for each sampled microhabitat: skin, soil, water, and matrix. The environmental variables tested included fragment area and perimeter, interfragment distance, distance to human dwellings, maximum, minimum and median temperatures of microhabitats, maximum, minimum and median relative humidity of microhabitats, pH, temperature, dissolved oxygen, conductivity, and total dissolved solids in bodies water. The RELATE test revealed correlation between these variables and the skin microbiota of *P. boiei* (Spearman, *ρ* = 0.398, *p* = .02), but only for unweighted UniFrac metric. The BEST analysis showed that this correlation is based mainly on the combination of variables that include temperature of microhabitat and distance to human dwelling (Spearman, *ρ* = 0.799, *p* = .016). There were no significant correlations between environmental variables and communities of water, soil, and matrix.

### 3.4 Antimicrobial activity of the skin microbiota

*Anti‐bacteria activity* – Of the 517 isolated bacterial strains, 483 were used in bioassays (34 of the original isolates did not remain viable). Results of the cross‐streak bioassay identified 84 bacterial isolates with antimicrobial activity against the growth of at least one of the twelve pathogenic bacteria strains. The percentage of isolates with antibacterial activity varied between sites, from 5.6% to 64% relative to the total isolates described (Appendix: Figure S1). Abundances ranged from 1 to 8 antibacterial isolates per individual frog. The total number of isolates and the total number of isolates with antimicrobial activity were positively and significantly related (Spearman, *ρ* = 0.82, *p* < .0001).

**Figure 7:**
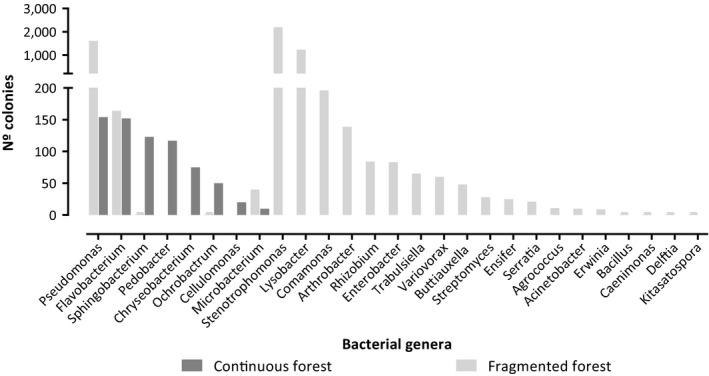
Taxonomic distribution and abundance of antimicrobial bacterial genera in continuous and fragmented forests

A total of 27 bacterial genera were identified from cultured isolates. The alpha diversity of antibacterial genera differed between the forest types for both Shannon and Simpson metrics (diversity *t* test, *p* < .05 for both) with fragments showing higher diversity. This result is congruent with that observed in the high‐throughput sequencing analyses, which showed that skin microbiota from *P. boiei* was different between populations from continuous and fragmented forests, with a greater diversity in the fragments. The genus *Pseudomonas* ranked highest in abundance abundances for both forest types. *Stenotrophomonas* was the second most abundant genus in the fragments, but it was not detected in the samples collected from continuous forests. Only five of the twenty‐seven genera with antimicrobial activity were detected in both forest types (Figure 7).

Besides being dominant, *Pseudomonas* was the genus with the greatest antimicrobial potential and inhibited the greatest number (ten out of twelve) of pathogenic bacterial strains. *Stenotrophomonas* and *Streptomyces* had the next greatest antimicrobial activity, inhibiting eight of twelve strains. *Arthrobacter*, *Delftia*, *Ensifer*, *Pedobacter,* and *Variovorax* each were effective against the growth of only one bacterial pathogen. Among these pathogenic strains, *A. hydrophila* showed sensitivity to the largest number, 74% of the total, of bacterial genera isolated from *P. boiei*. On the other hand, *E. coli* growth was affected only by the genus *Lysobacter* (Table 2).

**Table 2:** Range of antibacterial activity of bacterial genera isolated from skin microbiota of *Proceratophrys boiei*

Bacterial taxa	*Bd023*	*Bd423*	*Ah*	*Ml*	*Pa*	*Se*	*Sa*	*Pv*	*Ef*	*St*	*Kp*	*Ea*	*Se*	*Ec*	Total
*Pseudomonas*	+	+	+	+	+	+	+	+	+	+	+	+	+		13
*Stenotrophomonas*	+	+	+	+	+	+	+	+		+	+				10
*Streptomyces*	+	+	+	+	+	+	+	+	+			+			10
*Lysobacter*	+	+	+	+	+	+	+		+					+	9
*Sphingobacterium*	+		+	+	+	+	+		+	+			+		9
*Microbacterium*	+	+	+	+		+	+	+	+						8
*Ochrobactrum*	+	+	+	+	+	+				+			+		8
*Rhizobium*			+	+	+	+	+	+	+		+				8
*Agrococcus*			+	+	+	+				+	+	+			7
*Buttiauxella*	+	+	+		+				+		+		+		7
*Flavobacterium*	+	+	+	+	+			+							6
*Kitasatospora*	+	+	+	+		+	+								6
*Acinetobacter*	+		+	+		+	+								5
*Bacillus*				+		+	+			+					4
*Enterobacter*	+		+		+								+		4
*Erwinia*	+	+	+		+										4
*Trabulsiella*	+					+	+			+					4
*Arthrobacter*	+	+		+											3
*Caenimonas*				+	+							+			3
*Comamonas*	+				+			+							3
*Delftia*	+	+										+			3
*Variovorax*	+	+						+							3
*Cellulomonas*			+		+										2
*Chryseobacterium*			+					+							2
*Ensifer*		+		+											2
*Pedobacter*		+	+												2
*Serratia*	+		+												2
Total	19	15	19	16	15	13	11	9	7	7	5	5	5	1	

Note

*Bd*, *Batrachochytrium dendrobatidis*; *Ah*, *Aeromonas hydrophila*; *Ml*, *Micrococcus luteus*; *Pa*, *Pseudomonas aeruginosa*; *Se*, *Staphylococcus epidermidis*; *Sa*, *Staphylococcus aureus*; *Pv*, *Proteus vulgaris*; *Ef*, *Enterococcus faecalis*; *St*, *Salmonella sorovar Typhi*; *Kp*, *Klebsiella pneumoniae*; *Ea*, *Enterobacter aerogenes*; *Se*, *Salmonella enteritidis*; *Ec*, *Escherichia coli*. Obs.: Inhibition above 50% for *Bd* strains.

**Figure 8:**
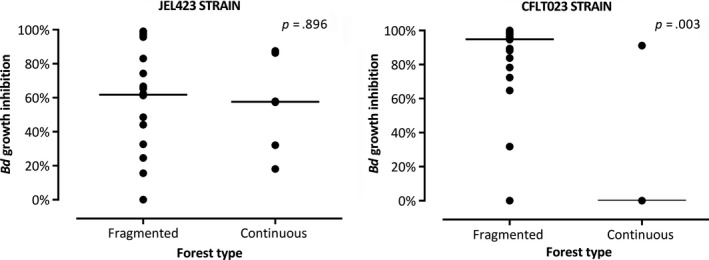
Growth inhibition in two strains of *Bd* caused by metabolites derived from cutaneous bacteria in *Proceratophrys boiei*, split by genera. Each point represents the mean of all isolates identified as belonging to a given genus

*Anti‐Bd activity* – The metabolites of 80 bacterial morphotypes that exhibited antimicrobial activity against pathogenic bacteria were tested against the growth of the pathogenic fungus *B. dendrobatidis*. This criterion selects skin bacteria with the best antimicrobial capacity. In total, 68 morphotypes exhibited some anti‐*Bd* activity against at least one of the *Bd* strains, and the potential of inhibition was comparable between strains (Mann–Whitney, *p* = .614), ranging from 15.6% to 99.1% in JEL423 and from 1.4% to 100% in CLFT023. However, forest type did have an effect on percent of inhibition, although only in CLFT023. Values were comparable for JEL423 (Mann–Whitney, *p* = .896) whereas the alternative strain displayed higher growth inhibition from metabolites isolated from bacteria in specimens from fragmented forests (Figure 8; Mann–Whitney, *p* = .003).

## 4 DISCUSSION

### 4.1 Microbial environmental sources

The present study shows that the bacterial communities of the microenvironments sampled from water, soil, skin, and matrix are different. Each microhabitat may serve as a selective filter that determines local microbial diversity, also known as the Baas‐Becking principle (Mizrahi‐Man, Davenport, & Gilad, 2013). While previous studies have demonstrated that the skin microbiota profile is influenced by the phylogenetic identity of the host amphibian (Muletz, Myers, Domangue, Herrick, & Harris, 2012), the current study shows that the bacterial communities in the microhabitats used by hosts also contribute to skin microbiota structure. This is especially important given that the soil is the primary habitat of *P. boiei* and clearly serves as the primary environmental source for this species' skin microbiota. Even though most individuals were collected and sampled during the reproductive season, when they spend significant time near water bodies, this species is essentially terrestrial (Pasteris, Bühler, & Nader‐Macías, 2006), and burrows into the soil during periods of low activity (pers. obs.).

### 4.2 Habitat quality and microbial communities

In the present study, the environmental bacterial communities of continuous forests and forest fragments did not differ. The result supports the hypothesis that microenvironments harbor specific communities, and environmental variation at a broader scale does not influence microbial communities. These data corroborate findings by Konopka (Piovia‐Scott et al., 2017), who reports that studying microbial communities at the macroscale is inadequate because the physicochemical characteristics of the occupied microenvironment are more relevant. Additionally, it is also worth emphasizing that the environmental variables in our study were linked already to the presence of individual *P. boiei*. From this perspective, these results involve the intentional comparison of microbial communities in nonrandom sampling sites identified by the presence of *P. boiei* in specific locations of the Atlantic Forest remnants. Although continuous and fragmented forests were not different in terms of significance, the higher values of environmental bacterial diversity in the fragments may reflect selective pressures that result from fragmentation. Land use influences the bacterial community structure of soil (Rocha et al., 2007); the fragments sampled in this study are, by definition, surrounded by human activity that includes crop and livestock, farming, and residential development.

Even in the absence of variation in the environmental microbial communities between continuous and fragmented forests, the *P. boiei* skin microbiota showed an effect of these type of forest. Changes in skin communities influenced by fragmentation cannot be interpreted, however, from the perspective of microhabitat since the microbial community of the soil, habitat used by *P. boiei*, did not exhibit significant differences between the two forest types. Thus, we can suggest that the observed differences in the abundance of bacteria of the cutaneous microbiota of this species seem to be more related to intrinsic characters of the host. For example, external factors, such as pollutants, stressors, or temperature and humidity regimes, which may alter the production of bioactive molecules in amphibian skin (Rollins‐Smith et al., 2002; Schadich & Cole, 2010; Simmaco et al., 1997). Recent reports indicate that the dynamic of the microbiome is regulated by the density of microbial life on the host (Toledo, Britto, Araújo, Giasson, & Haddad, 2006), which is important from a conservation standpoint. The abundance of bacterial populations should be considered not only in terms of the structure of the cutaneous microbiota, but also in terms of its function and relationship with the host (Walke, Harris, Reinert, Rollins‐Smith, & Woodhams, 2011). On *P. boiei* skin, composition may be a more resilient aspect of cutaneous bacterial communities.

### 4.3 Microbial communities and abiotic variables

The present study demonstrated a correlation between the thermal profile of the microhabitats occupied by *P. boiei* and the composition of its cutaneous bacterial community. This relationship can be explained by the ectothermic nature of frogs. Amphibian skin characteristics are affected by environmental temperature, and a recent study indicated that the diversity of the skin microbiota can be affected by body temperature of the amphibian host (Wang, Garrity, Tiedje, & Cole, 2007). The influence of landscape on this relationship should also be considered, at least in terms of abiotic variables, given that the continuous forests and fragments differ substantially in thermal profiles. Individuals of *P. boiei* are exposed to lower temperature in the continuous forests, such as Cunha and Santa Virgínia compared to those in areas with greater fragmentation (unpublished data).

### 4.4 Antimicrobial activity of the skin microbiota

This study demonstrates that bacterial strains with antimicrobial activity are part of the cutaneous microbiota of *P. boiei*. If our in vitro experiments reflect realistic phenomena in natural environments, the cutaneous microbiota of *P. boiei* serves as an important component in the prevention of infectious diseases, especially given that all individuals tested harbor bacteria capable of inhibiting growth of pathogens. Another important observation was that higher bacterial richness is related to a higher number of bacteria with antimicrobial activity, and amphibians living in fragmented forests harbor higher diversity. This pattern can also have value for population health because there is a correlation between bacterial richness of the amphibian microbiota and its efficacy against *Bd* (Crawshaw & Fowler, 1993; Weisburg, Barns, Pelletier, & Lane, 1991). The broad spectrum of action observed for the genera *Pseudomonas*, *Stenotrophomonas*, *Streptomyces*, *Lysobacter*, *Sphingobacterium*, *Microbacterium*, *Ochrobactrum,* and *Rhizobium*, and effectiveness against pathogenic fungi and bacteria indicate a broad spectrum of antimicrobial activity. It is worth mentioning that most of the antimicrobial genera (or corresponding family) were detected in the core diversity of *P. boiei* cutaneous microbiota (Appendix: Table S1) and may be related to important functions for the animal (Toledo et al., 2006; Williston, Zia‐Walrath, & Youmans, 1947).

We found evidence that frog populations from the two habitat types differ in the bacteria they harbor that inhibit growth of pathogens, and we conclude that forest type influences diversity of anti‐pathogenic bacteria harbored by *P. boiei*, and perhaps also other species. In addition, differences also emerged in the anti‐*Bd* activity of *P. boiei* skin microbiota, but, interestingly, this difference applies only for the Brazilian strain of *Bd*. Previous studies demonstrate prevalence of anti‐*Bd* bacteria in natural populations of amphibians, which may help populations persist during local chytrid fungus epidemics (Brockett et al., 2012; Burkey, 2013). Given that *Bd* presence has been documented for our study area (Woodhams et al., 2007), the results reported may be partially explained by a history of contact with this fungus. However, there is no information about the current prevalence of this fungus in the continuous and fragmented forests. Despite this limitation, differences in resistance to chytrid fungus among frog populations have been attributed to the composition and metabolites of their cutaneous microbiota (Yasumiba, Bell, & Alford, 2016) and population differences in the skin microbiota reported here may help explain differential vulnerability to fungus and other pathogens.

Preserving and maintaining the population dynamics of well‐established skin microbiomes may be the best strategy to prevent pathogenic infections in amphibians. Even so, the magnitude of these differences is notable: in the case of *P. boiei*, differences exist among populations but the presence of bacteria with antimicrobial activity in all tested individuals may be enough to inhibit colonization by pathogenic microorganisms. The results of this study indicate that the sustainability of amphibian populations may be related to the interactions among host, microorganisms, and environment.

## CONFLICT OF INTEREST

The authors declare no conflict of interest.

## AUTHOR CONTRIBUTIONS

AB and CA designed the study. AB collected all field data. AB, CR, and CC did the laboratory work. AB, CR, and CA did the statistics analysis. AB wrote the paper. All authors contributed to revision of the manuscript and approved the submitted material.

## Data Availability

The 16S rRNA gene sequences of the culturable bacterial strains were deposited at GenBank under accession numbers MG008695–MG008767 and MH751912–MH751922. The sequences from the metagenomic analysis were deposited at MG‐RAST version 4.0.3 under the accession numbers mgm4798021.3 through mgm4798052.3.
