# Supplementary material for: Environmental influences on and antimicrobial activity of the skin microbiota of Proceratophrys boiei (Amphibia, Anura) across forest fragments
Source: Ecol Evol. 2020 Jan 7;10(2):901–13. doi: 10.1002/ece3.5949 (PMC6988551; doi:10.1002/ece3.5949)
Supplement: Supplementary file 1 [file ECE3-10-901-s001.pdf]

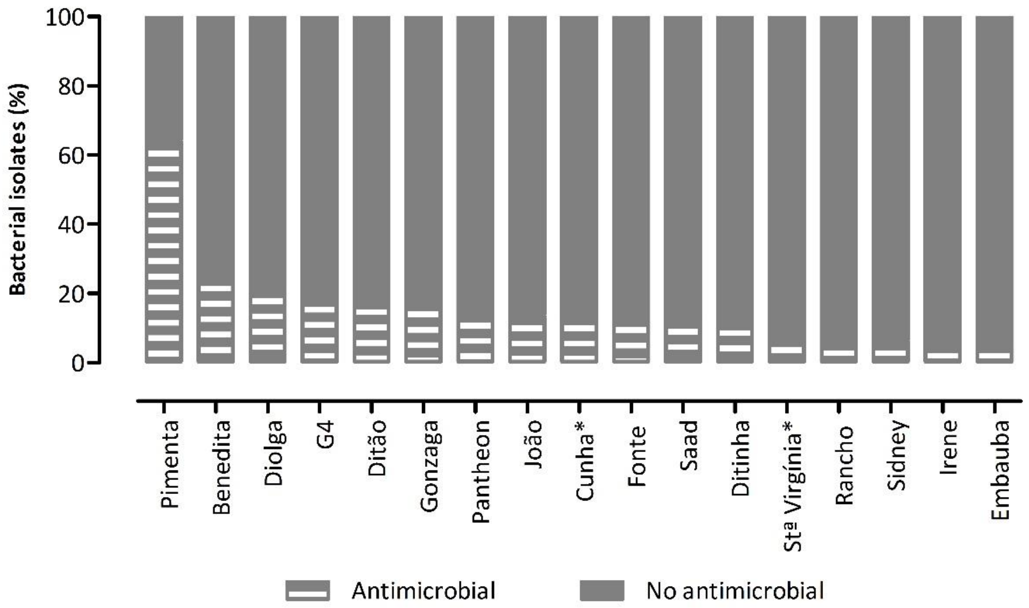

Appendix Figure 1: Distribution of bacterial isolates of *P. boiei* skin microbiota with and without antimicrobial activity by each local. *Continuous forests. Note: This data set does not include the metabolite bioassay against *Bd*.

Appendix Table 1: Taxonomic profile of core skin microbiota composition at the family and genus level for *P. boiei* skin. Core OTUs occurs in all samples assessed.

**Family**	**Genus**	**Total of OTUs**
Acetobacteraceae		1
Aurantimonadaceae		1
Bacillaceae		2
Bacillaceae	*Bacillus*	1
Beijerinckiaceae		5
Beijerinckiaceae	*Beijerinckia*	1
Bradyrhizobiaceae		13
Bradyrhizobiaceae	*Bosea*	2
Bradyrhizobiaceae	*Bradyrhizobium*	1
Brucellaceae	*Ochrobactrum*	1
Burkholderiaceae		5
Burkholderiaceae	*Burkholderia*	4
Caulobacteraceae		3
Caulobacteraceae	*Mycoplana*	1
Chitinophagaceae		3
Chthoniobacteraceae		2
Chthoniobacteraceae	*Xiphinematobacter*	1
Comamonadaceae		22
Comamonadaceae	*Methylibium*	4
Comamonadaceae	*Roseateles*	2
Conexibacteraceae		1
Cytophagaceae	*Dyadobacter*	2
Enterobacteriaceae		5
Flavobacteriaceae	*Flavobacterium*	1
Frankiaceae		8
Gaiellaceae		3
Hyphomicrobiaceae		33
Hyphomicrobiaceae	*Rhodoplanes*	22
Hyphomicrobiaceae	*Hyphomicrobium*	5
Hyphomicrobiaceae	*Devosia*	4
Hyphomonadaceae		1
Intrasporangiaceae		5
Intrasporangiaceae	*Phycicoccus*	3
Isosphaeraceae		1
Kineosporiaceae		3
Kineosporiaceae	*Kineococcus*	1
Methylobacteriaceae	*Methylobacterium*	3
Methylocystaceae		5
Methylocystaceae	*Methylopila*	1
Microbacteriaceae		14
Microbacteriaceae	*Leucobacter*	3
Microbacteriaceae	*Microbacterium*	3

cont.

**Family**	**Genus**	**Total of OTUs**
Microbacteriaceae	*Rathayibacter*	3
Microbacteriaceae	*Agrococcus*	1
Microbacteriaceae	*Curtobacterium*	1
Microbacteriaceae	*Salinibacterium*	1
Micromonosporaceae		15
Micromonosporaceae	*Actinoplanes*	4
Micromonosporaceae	*Dactylosporangium*	1
Mycobacteriaceae	*Mycobacterium*	19
Nakamurellaceae		1
Nocardioidaceae		34
Nocardioidaceae	*Nocardioides*	19
Patulibacteraceae		1
Phyllobacteriaceae		7
Phyllobacteriaceae	*Aminobacter*	1
Phyllobacteriaceae	*Mesorhizobium*	1
Pirellulaceae		1
Planctomycetaceae	*Planctomyces*	2
Propionibacteriaceae	*Propionibacterium*	1
Pseudomonadaceae		3
Pseudomonadaceae	*Pseudomonas*	2
Pseudonocardiaceae	*Pseudonocardia*	3
Pseudonocardiaceae	*Actinomycetospora*	2
Rhizobiaceae		27
Rhizobiaceae	*Agrobacterium*	10
Rhizobiaceae	*Rhizobium*	1
Rhizobiaceae	*Shinella*	1
Rhodobacteraceae	*Rhodobacter*	1
Rhodospirillaceae		6
Sinobacteraceae		3
Sphingobacteriaceae		4
Sphingobacteriaceae	*Pedobacter*	1
Sphingomonadaceae		21
Sphingomonadaceae	*Sphingomonas*	9
Sphingomonadaceae	*Novosphingobium*	2
Sphingomonadaceae	*Kaistobacter*	1
Sphingomonadaceae	*Sphingobium*	1
Streptomycetaceae		4
Streptomycetaceae	*Streptomyces*	2
Xanthobacteraceae	*Labrys*	2
Xanthomonadaceae		6
Xanthomonadaceae	*Stenotrophomonas*	3
Xanthomonadaceae	*Luteibacter*	2
